# Navigating Uncertainty in Pediatric Chronic Illness Care: A Hermeneutic Phenomenological Study of Nurses in Saudi Arabia

**DOI:** 10.3390/healthcare14152392

**Published:** 2026-08-04

**Authors:** Abdulaziz M. Alodhailah, Rehan Eid, Bader M. Almutairy, Faihan F. Alshaibany, Hend N. Almutairi, Mohammed Almutairi

**Affiliations:** 1 Department of Medical-Surgical Nursing, College of Nursing, King Saud University, Riyadh 11451, Saudi Arabia; 2 King Abdullah Medical Complex—Maternity and Children Specialized Hospital, Jeddah 23816, Saudi Arabia; 3 Department of Community and Mental Health Nursing, College of Nursing, King Saud University, Riyadh 11451, Saudi Arabia; 4 Department of Nursing Administration and Education, College of Nursing, King Saud University, Riyadh 11451, Saudi Arabia; 5 School of Archaeology and Anthropology, College of Arts and Social Sciences, The Australian National University, Canberra, ACT 2601, Australia

**Keywords:** pediatric nursing, complex chronic conditions, clinical uncertainty, qualitative research, coping, lived experience

## Abstract

**Background/Objectives:** Children with complex chronic conditions (CCCs) present profound clinical uncertainty for pediatric nurses, who must navigate unpredictable disease trajectories, ambiguous symptom presentations, and emotionally demanding care situations. This study explored the lived experiences of pediatric nurses caring for children with CCCs, with a specific focus on how they perceive, navigate, and manage clinical and emotional uncertainty in daily practice. **Methods:** A Gadamerian hermeneutic phenomenological study was conducted in pediatric units within tertiary hospitals in Riyadh, Saudi Arabia, using purposive, maximum-variation sampling. Fifteen pediatric nurses participated in semi-structured interviews conducted in English. Data were analysed using hermeneutic phenomenological analysis informed by the framework of Alsaigh and Coyne. Reporting followed the Consolidated Criteria for Reporting Qualitative Research (COREQ) guidelines. **Results:** Three themes emerged: navigating the unknown, reflecting nurses’ encounters with diagnostic ambiguity and unpredictable illness trajectories; the emotional weight of not knowing, capturing the psychological burden of sustained uncertainty; and finding footing in uncertainty, describing adaptive strategies including relational anchoring, peer support, and meaning-making through caregiving. **Conclusions:** Clinical uncertainty is an inherent and pervasive feature of pediatric chronic illness care. Pediatric nurses develop relational and meaning-making strategies, alongside more limited cognitive appraisal strategies, to manage uncertainty, yet require greater institutional recognition and support. Targeted interventions addressing uncertainty tolerance, reflective practice, and structured peer support are proposed as potential approaches, warranting future evaluation, to strengthen nursing resilience and support care quality for children with CCCs.

## 1. Introduction

Children with complex chronic conditions (CCCs) represent a growing and clinically significant population in pediatric healthcare worldwide [1]. Advances in medical technology, neonatal intensive care, and disease-modifying therapies have extended survival for children with previously life-limiting diagnoses, including congenital anomalies, neurodegenerative disorders, metabolic diseases, and childhood cancers requiring prolonged treatment [2]. In this study, complex chronic conditions were operationally defined as conditions expected to last at least twelve months, involving multiple organ systems or a single system severely enough to require specialist paediatric care, and typically overlapping with, though not synonymous with, the broader category of children with medical complexity. While these advances represent important achievements, they have simultaneously increased the complexity, chronicity, and unpredictability of illness trajectories, creating substantial care challenges for pediatric nurses who provide continuous bedside assessment and holistic support [3].

Clinical uncertainty is recognised as a fundamental feature of healthcare, yet its impact on nursing practice in pediatric chronic illness care remains underexplored [4]. Uncertainty in this context encompasses diagnostic ambiguity, prognostic unpredictability, fluctuating symptom profiles, and the difficulty of interpreting clinical cues in children whose baseline presentations are already complex and atypical [5]. Unlike acute illness scenarios where deterioration follows relatively predictable physiological trajectories, CCCs often involve prolonged periods of clinical ambiguity during which nurses must sustain attentiveness, make judgements with incomplete information, and communicate concern in the absence of definitive evidence [6].

Mishel’s Uncertainty in Illness Theory provides a foundational framework for understanding how individuals appraise and respond to illness-related uncertainty [7]. Originally developed for patients and families, the theory posits that uncertainty arises when events cannot be adequately structured or categorised due to insufficient information, complexity, or unpredictability. Although developed to describe patients’ and families’ appraisal of illness, this cognitive-appraisal structure has been extended in the nursing literature to describe clinicians’ own uncertainty when faced with ambiguous or incomplete clinical information, providing an adapted rather than directly transposed lens for the present study of nurses. When applied to nursing practice, uncertainty is conceptually understood as a multidimensional cognitive-appraisal process [8]. Uncertainty, moral distress, and moral residue are conceptually distinct: moral distress arises specifically when a nurse knows the ethically appropriate action but is constrained from taking it, whereas uncertainty reflects an absence of clear knowledge about the clinical situation itself. The two constructs may nonetheless intersect in practice, as repeated, unresolved uncertainty can contribute to conditions under which moral distress and its cumulative form, moral residue, develop [9]. Pediatric nurses are uniquely positioned at the intersection of clinical ambiguity and relational care, often serving as primary interpreters of subtle changes while simultaneously managing the emotional needs of children and their families [10].

The emotional dimension of uncertainty in pediatric nursing practice is increasingly acknowledged in the literature. Caring for children with uncertain prognoses can generate anticipatory grief, compassion fatigue, and feelings of professional inadequacy [11,12]. Nurses may experience moral distress when institutional expectations demand definitive clinical decisions despite incomplete knowledge, or when care goals conflict between curative and palliative orientations [13]. These experiences are compounded in chronic illness care, where parents’ own hope-related emotional responses to prognostic uncertainty shape the relational demands placed on nurses, deepening emotional investment and heightening the personal impact of clinical ambiguity [14].

Despite growing recognition of uncertainty as a professional challenge, existing research has predominantly focused on uncertainty from patient and family perspectives, or on its role in acute, time-limited clinical decisions [15,16]. Less attention has been paid to how pediatric nurses experience sustained uncertainty in the context of chronic conditions, where ambiguity persists across weeks, months, or years of care. Furthermore, nurses’ capacity to perceive and respond to clinical ambiguity develops through situated practice and experiential expertise within particular institutional contexts [17], and these institutional and cultural contexts jointly shape how uncertainty is experienced and managed. In Saudi Arabia, where nursing practice is situated within a rapidly transforming healthcare system guided by Vision 2030, pediatric nurses navigate uncertainty amidst evolving professional expectations, cultural norms surrounding childhood illness, and institutional demands for standardised care delivery [18,19]. These features may shape how uncertainty is disclosed to families, how it is discussed within multidisciplinary and hierarchical teams, and how far individual nurses feel able to voice ambiguity to supervisors and colleagues from different professional and national backgrounds, although the present study’s evidence on this point is necessarily indirect (see Section 4.1).

Understanding the lived experience of pediatric nurses coping with uncertainty is essential for developing targeted educational interventions, supportive workplace structures, and clinical frameworks that acknowledge the interpretive and emotional labour inherent in caring for children with CCCs. The aim of this study was therefore to explore how pediatric nurses experience and manage clinical and emotional uncertainty when caring for children with complex chronic conditions. Specifically, the study sought to address the following research questions:

1. How do pediatric nurses describe their experiences of clinical uncertainty when caring for children with CCCs?
2. What cognitive, emotional, and relational strategies do nurses employ to navigate sustained uncertainty?
3. How are institutional and cultural contextual factors reflected within pediatric nurses’ accounts of navigating and coping with uncertainty in daily practice?

Because institutional and cultural context is explored as an interpretive lens running across the three themes reported in Section 3.2, rather than through a single dedicated theme, Research Question 3 is answered here through participants’ relational and emotional accounts of everyday practice rather than through direct evidence on system-level policy or organisational structures; this narrowed scope is made explicit in Section 3.2, and the corresponding limitation is discussed in Section 4.1.

## 2. Materials and Methods

This study was situated within a constructivist–interpretivist paradigm, which holds that meaning is co-constructed between researcher and participant, and that lived experience can only be adequately understood within its historical, cultural, and relational context. Consistent with this paradigm, an interpretive (hermeneutic) phenomenological design was employed to explore how pediatric nurses experience and navigate uncertainty when caring for children with complex chronic conditions (CCCs). This study adopted a single, coherent hermeneutic phenomenological framework: Heideggerian ontology, which understands lived experience as always already situated within a historical, cultural, and relational world, provided the philosophical foundation, while Gadamerian hermeneutics, elaborated methodologically for health research by Alsaigh and Coyne, provided the specific analytic method used to move between data collection and interpretation [20], consistent with the broader aim of hermeneutic phenomenology to understand lived experience as situated within its historical, cultural, and relational context rather than as an isolated cognitive phenomenon [21]. This methodology was judged appropriate because coping with uncertainty involves tacit knowledge, emotional processing, relational engagement, and contextual interpretation that extend beyond measurable clinical indicators, and because the hermeneutic circle, the iterative movement between individual accounts, researcher pre-understanding, and the emerging interpretive whole, provided an explicit analytic mechanism for developing interpretive, rather than purely descriptive, findings. The research aims, research questions, sampling strategy, data-collection method, and analytic approach were maintained in alignment throughout the study: the research questions asked how nurses experience and cope with uncertainty, and how institutional and cultural context is reflected within their accounts of doing so; purposive sampling targeted nurses with direct, ongoing exposure to this phenomenon; semi-structured in-depth interviews were used to generate rich first-person accounts; and hermeneutic analysis was used to interpret these accounts without reducing them to categorical summaries. The study is reported in accordance with the Consolidated Criteria for Reporting Qualitative Research (COREQ) 32-item checklist [22]; the completed checklist is provided as Supplementary File S2.

### 2.1. Study Setting

The study was conducted in pediatric medical, surgical, and oncology units within tertiary healthcare hospitals in the Riyadh region, Saudi Arabia. These hospitals serve as referral centres for complex pediatric conditions, including congenital heart disease, childhood cancers, neurological disorders, metabolic conditions, and chronic respiratory illnesses. The units provide specialised services including continuous monitoring, multidisciplinary care coordination, palliative care consultations, and family-centred care programmes. The institutional context, characterised by protocol-driven care pathways and a multinational nursing workforce drawn from Saudi and expatriate backgrounds, provided a relevant setting for exploring how cultural, linguistic, and professional factors shape nurses’ experiences of uncertainty. No member of the research team held a supervisory, managerial, or performance-evaluation role over potential participants at the time of the study, and data collection was conducted independently of routine clinical operations and staff appraisal processes to minimise real or perceived pressure to participate.

### 2.2. Participants and Sampling

Participants were registered nurses working in pediatric units with direct responsibility for the ongoing care and assessment of children with complex chronic conditions. Inclusion criteria were: (1) at least one year of pediatric nursing experience; (2) current clinical involvement in direct patient care; and (3) experience caring for children with diagnosed CCCs, including, but not limited to, congenital anomalies, chronic respiratory conditions, oncological diagnoses, or neurodevelopmental disorders. Nurses in purely administrative or supervisory roles without bedside responsibilities were excluded.

Purposive, maximum-variation sampling was used to recruit participants who could provide rich, experience-based accounts of navigating uncertainty. Efforts were made to achieve variation in years of experience, educational background, nationality, and clinical subspecialty to enhance the depth and transferability of findings. Sample size was determined prospectively using the concept of information power [23] rather than a numerical saturation target [24]; because the study had a narrowly defined aim, a specific and relatively homogeneous participant group, an established interpretive theoretical framework, high-quality in-depth dialogue, and a case-by-case hermeneutic analysis strategy, a comparatively small sample was expected to carry sufficient information power. An anticipated range of 12–16 nurses was set a priori; recruitment continued until the research team judged, through ongoing analytic discussion conducted in parallel with data collection, that further interviews were unlikely to substantially alter the interpretive account, consistent with the concept of interpretive sufficiency in hermeneutic-phenomenological research [20]. The decision to stop recruitment was therefore an information-power judgement about the sufficiency and quality of the accumulated interpretive material, made jointly by the research team, rather than a count-based test of thematic repetition; the range of 12–16 nurses served only as an a priori planning estimate, not a saturation target. A final sample of 15 nurses was recruited (Table 1). A total of 20 nurses were approached and invited to participate; of these, 2 declined, 2 did not respond, and 1 withdrew before their scheduled interview, most commonly citing a busy work schedule as the reason.

### 2.3. Recruitment

Following institutional approvals, the research team informed pediatric unit nurse managers about the study; nurse managers assisted only in circulating a study invitation by email and played no role in selecting, screening, or personally approaching potential participants. Interested nurses contacted the research team directly, by email or telephone, to express interest and arrange an interview; this indirect recruitment route was chosen specifically to minimise perceived managerial influence on participation decisions. No member of the research team had a pre-existing professional or personal relationship with any participant before the study. Participants were informed of the researcher’s occupation, position, and reasons for conducting the research prior to their interview. Written information sheets outlining the study purpose, procedures, voluntary participation, confidentiality measures, data-security arrangements, and withdrawal rights were provided to all potential participants before written informed consent was obtained.

### 2.4. Data Collection

Data were collected through individual, semi-structured, in-depth interviews conducted between April and May 2026 across three tertiary healthcare hospitals in the Riyadh region of Saudi Arabia. Each participant was interviewed once, with no repeat interviews undertaken. Interviews were conducted either face-to-face in private, closed-door rooms within the participating institutions (*n* = 6) or via secure, encrypted online video conferencing platforms when in-person meetings were not feasible (*n* = 9). No individuals other than the participant and the interviewer were present during any interview. The duration of interviews ranged from 44 to 65 min (mean duration: 53.2 ± 6.1 min), with a total interview time of 798 min across the full sample (see Section 3.1).

An interview guide was developed specifically for this study based on existing literature on clinical uncertainty, moral distress, pediatric nursing practice, and the study’s conceptual framework. The guide was pilot-tested with four pediatric nurses who were not included in the final sample to assess the clarity, relevance, and flow of the questions prior to data collection. Following pilot testing, minor wording modifications were made to improve the clarity and comprehensibility of the interview questions, while the overall structure and content of the guide remained unchanged. Consistent with English’s role as the primary working language in multinational tertiary hospital settings in Saudi Arabia, all interviews were conducted, audio-recorded, and transcribed in English; no interviews were conducted in Arabic or another language, and no translation was undertaken at any stage of data collection, transcription, or analysis. The English version of the semi-structured interview guide is provided as Supplementary File S1. Questions were open-ended and exploratory, encouraging participants to reflect on concrete clinical experiences. Example prompts included:

“Can you describe a situation where you felt uncertain about a child’s condition or how to manage their care?”

“What was that experience like for you emotionally?”

“How did you decide what to do when the clinical picture was unclear?”

“How did you communicate your concerns to the medical team or the family?”

“What helps you cope when you face ongoing uncertainty in your work?”

Probing questions were used to elicit clarification and deepen reflection on the cognitive, emotional, relational, and contextual dimensions of uncertainty. The interview guide evolved iteratively to explore emerging insights while maintaining consistency across interviews. All interviews were audio-recorded with participant consent and transcribed verbatim by a trained member of the research team. Transcripts were checked against the original audio recordings for accuracy before analysis.

Transcripts were not returned to participants for correction. Instead, a summary of the preliminary themes was shared with three participants (20% of the sample) as part of the credibility procedures described in Section 2.7. These participants were purposively selected to reflect diversity within the sample in terms of clinical unit, years of pediatric nursing experience, and professional background. All three participants provided feedback on the preliminary findings. Their comments primarily confirmed the accuracy and relevance of the interpretations and provided minor clarifications regarding the wording and contextual description of some themes. No major changes were required; however, minor refinements were made to improve the clarity and contextual accuracy of the final thematic analysis.

Field notes were completed immediately after each interview to document contextual observations, non-verbal cues, and the interviewer’s preliminary interpretive reflections. These notes were incorporated into the audit trail and informed subsequent interviews and the analytical process.

### 2.5. Researcher Characteristics and Reflexivity

Interviews were conducted by the first author, an academic nurse with clinical experience in pediatric and critical care nursing and a scholarly background in qualitative and geriatric research. No member of the research team had a prior personal or clinical relationship with any participant, and participants had no prior knowledge of the interviewer beyond their general professional role within the healthcare system. Given this positioning, reflexive awareness was central to the research process. Hermeneutic phenomenology acknowledges that researchers enter inquiry with prior understandings and assumptions that shape interpretation; rather than attempting to bracket preconceptions entirely, the researcher engaged in reflexive journaling before, during, and after data collection and analysis to critically examine assumptions about uncertainty, coping, and childhood chronic illness, and to consider how the researcher’s own clinical background might shape the interpretation of participants’ accounts. Regular analytic discussions were conducted within the research team to challenge emerging interpretations, surface alternative readings of the data, and minimise unexamined bias; disagreements in interpretation were resolved through discussion and, where necessary, re-engagement with the original transcripts.

### 2.6. Data Analysis

Data analysis followed the principles of hermeneutic phenomenological interpretation, informed by the framework of Alsaigh and Coyne [20] for conducting Gadamerian hermeneutic analysis. Two members of the research team independently coded a subset of transcripts before comparing and reconciling their coding, in order to enhance analytic rigour and reduce individual interpretive bias. Two coders in total conducted the analysis, with two independent rounds of audit to reconcile discrepancies and reach consensus on the coding. Analysis began with immersion in the transcripts through repeated reading to gain a holistic sense of each participant’s experience. Initial meaning units were identified line-by-line, focusing on descriptions of encountering, interpreting, and managing uncertainty in pediatric chronic illness care, and were assigned preliminary codes.

The analysis proceeded through a hermeneutic circle, moving iteratively between individual accounts, codes, and the emerging interpretive whole to refine understanding. Related codes were grouped into candidate sub-themes, which were then reviewed against the full data set and refined into the final themes and sub-themes presented in Section 3.2. Patterns across participants were explored to identify shared experiential structures while preserving contextual nuance and divergent accounts. Themes were not treated as categorical summaries but as interpretive constructions that illuminated the underlying meanings of uncertainty and coping. Analytic decisions were documented in a detailed audit trail, including coding notes, theme development memos, and records of team discussions, to support dependability and confirmability. Data management, coding, and theme development were supported using NVivo 14 (QSR International) to organise transcripts, store codes, and facilitate systematic comparison across cases.

### 2.7. Rigour and Trustworthiness

Rigour was established through credibility, dependability, confirmability, and transferability. Credibility was enhanced through prolonged engagement with the data, iterative questioning during interviews, and peer debriefing within the research team. Selected participants were invited to review a summary of emerging themes to ensure that interpretations resonated with their experiences. Dependability was supported by maintaining a transparent audit trail of analytic decisions. Confirmability was strengthened through reflexive journaling and team-based interpretation to ensure findings were grounded in participant narratives. Transferability was facilitated by providing detailed contextual descriptions of the setting, participants, and analytic processes, enabling readers to assess relevance to other pediatric care environments.

### 2.8. Ethical Considerations

Ethical approval for this study was obtained from the Institutional Review Board of King Saud University, Riyadh, Saudi Arabia (Reference No: 26-0329, on 1 April 2026), and from the participating hospitals where required. The study was conducted in accordance with the Declaration of Helsinki and relevant institutional requirements. Participation was voluntary, and written informed consent was obtained from all participants before their interview; consent covered audio-recording, verbatim transcription, and the use of anonymised quotations in publications. Participants were informed of their right to withdraw at any time, without consequence for their employment or clinical duties. Confidentiality was maintained by removing identifying information from transcripts, assigning pseudonyms in reporting, and storing audio files, transcripts, and consent forms on a password-protected, encrypted institutional server accessible only to the research team.

## 3. Results

### 3.1. Characteristics of Participants

A total of 15 pediatric nurses participated in the study. Participants were recruited from pediatric medical, surgical, and oncology units within tertiary healthcare hospitals in the Riyadh region, Saudi Arabia. The majority were female (*n* = 11, 73.3%), with ages ranging from 25 to 42 years. The mean age was 32.4 (±5.1) years. Most participants held a bachelor’s degree in nursing (*n* = 11, 73.3%), while four nurses (26.7%) had completed postgraduate education (master’s degree or specialty certification in pediatric nursing). Participants’ total nursing experience ranged from 2 to 18 years, with a mean of 8.7 (±4.5) years. Pediatric-specific experience ranged from 1 to 14 years, with a mean of 5.9 (±3.2) years. All participants had direct responsibility for ongoing assessment and care of children with complex chronic conditions. The total interview time was 798 min, with individual interviews lasting between 44 and 65 min (mean 53.2 ± 6.1 min). Participant characteristics are summarised in Table 1.

### 3.2. Thematic Findings

Hermeneutic analysis yielded three interrelated themes that capture how pediatric nurses experienced and coped with uncertainty when caring for children with complex chronic conditions. These themes reflect the cognitive burden of ambiguity, the emotional toll of sustained unknowing, and the adaptive strategies nurses developed to maintain professional functioning and compassionate engagement. Institutional and cultural context, including hierarchical communication norms, staffing and workload structures, and culturally shaped expectations surrounding childhood illness and family involvement, is not presented as a separate, fourth theme; rather, it runs through the three themes below as an interpretive lens, most directly visible in nurses’ accounts of relational anchoring (Theme 3.2.3) and of moral tension within hierarchical teams (Theme 3.2.2). Uncertainty was not described as a discrete event but as a persistent experiential condition woven into daily practice. Table 2 summarises the themes, sub-themes, and brief descriptions.

#### 3.2.1. Navigating the Unknown

This theme reflects how pediatric nurses experienced ongoing clinical uncertainty as a defining feature of caring for children with complex chronic conditions. Unlike acute care scenarios in which ambiguity is typically time-limited and resolved through diagnostic investigation, nurses described uncertainty as persistent, layered, and resistant to definitive resolution. Navigating the unknown involved sustained cognitive engagement with incomplete information, recurrent appraisal of ambiguous clinical cues, and the challenge of planning care within inherently unpredictable illness trajectories.

##### Living with Diagnostic Ambiguity

Participants described caring for children whose diagnoses were uncertain, evolving, or contested among medical specialists. This diagnostic ambiguity created a pervasive sense of operating without a clear clinical map.

*“Sometimes you are caring for a child, and nobody can tell you exactly what is wrong. The specialists disagree; the tests are inconclusive. You just keep going without a clear answer.”*

*(N3, Pediatric Medical, 7 years)*

Nurses described this ambiguity as particularly distressing when communicating with families who sought definitive explanations.

*“The parents ask, ‘What does my child have?’ and you cannot give them a straight answer. You feel inadequate, even though you know it is not your fault.”*

*(N8, Pediatric Oncology, 9 years)*

Several participants noted that diagnostic ambiguity was compounded by the rarity of certain conditions, leading to limited clinical guidelines and few precedents for care decisions.

*“Some of these children have conditions so rare that even the textbooks do not help you. You are making decisions based on very little evidence.”*

*(N12, Pediatric Surgical, 6 years)*

##### Unpredictable Illness Trajectories

Beyond diagnostic uncertainty, nurses described the unpredictability of disease progression as a major source of clinical difficulty. Children with CCCs often exhibited fluctuating courses characterised by unexpected improvements, sudden deteriorations, or prolonged plateaus that defied prognostic expectations.

*“One day the child seems better, responding well, and you feel hopeful. The next day, everything changes. You learn not to trust stability.” *

*(N1, Pediatric Medical, 10 years)*

This unpredictability challenged clinical planning and made it difficult for nurses to prepare emotionally for outcomes.

*“You cannot plan for the future because you do not know what tomorrow looks like. Will this child go home? Will they need more surgery? You just do not know.” *

*(N6, Mixed Pediatric, 4 years)*

Participants described developing a cautious optimism, balancing hope with preparedness for decline.

*“You hope for the best but prepare for the worst. That is how you survive in this work.” *

* (N14, Pediatric Oncology, 12 years)*

#### 3.2.2. The Emotional Weight of Not Knowing

This theme captures the psychological and moral burden of sustained uncertainty on pediatric nurses. Uncertainty was not experienced solely as a cognitive challenge but as an emotionally taxing condition that permeated professional identity and personal well-being. Nurses described carrying the emotional weight of ambiguity, often without adequate institutional outlets for processing these experiences.

##### Carrying the Burden Silently

Participants frequently described internalising the distress generated by clinical uncertainty, feeling that professional expectations demanded composure and confidence even when they felt unsure.

*“You cannot show the family that you are worried or confused. You have to appear strong, even when inside you are full of doubt.” *

*(N5, Pediatric Medical, 5 years)*

This emotional suppression was described as cumulative, with nurses noting that sustained uncertainty eroded their emotional reserves over time.

*“It builds up. Every child you care for where you do not know what will happen takes something from you. After a while, you feel exhausted but you cannot explain why.”*

*(N9, Pediatric Surgical, 11 years)*

Several participants described a sense of isolation, feeling that their emotional struggles were invisible to colleagues and supervisors.

*“Nobody asks how you are feeling. They assume you are coping because you keep working. But sometimes you are just going through the motions.”*

*(N2, Pediatric Medical, 3 years)*

##### Moral Tension Between Action and Restraint

Uncertainty frequently generated moral tension for nurses: this is interpreted here as moral, rather than purely clinical, ambivalence because participants framed the conflict in terms of competing obligations, to act protectively and to avoid inflicting harm through premature intervention, rather than as simple uncertainty about the correct clinical technique. Nurses described conflict between the professional imperative to act decisively and the recognition that premature or uninformed action could cause harm.

*“You want to do something, anything, to help the child. But sometimes the best thing is to wait and watch. That waiting is the hardest part.”*

*(N7, Pediatric Oncology, 8 years)*

This tension was particularly acute when families pressured nurses for definitive answers or rapid interventions.

*“The mother says, ‘Do something!’ and you understand her pain. But you also know that acting without enough information can make things worse.” *

*(N11, Pediatric Surgical, 10 years)*

Participants described this moral tension as a source of ongoing professional discomfort that was rarely acknowledged in formal training or institutional support structures.

*“We are taught to be decisive, to have answers. Nobody teaches you how to sit with not knowing.” *

*(N4, Mixed Pediatric, 6 years)*

#### 3.2.3. Finding Footing in Uncertainty

This theme explores the adaptive strategies nurses developed to manage uncertainty and sustain professional functioning. Despite the challenges described in previous themes, participants demonstrated resilience and resourcefulness in constructing approaches to cope with ambiguity. These strategies were relational, cognitive, and existential in nature.

##### Relational Anchoring

Relationships with colleagues and children emerged as primary resources for navigating uncertainty; relationships with families were also relevant but are represented more thinly in the data below, and this sub-theme should be read as a colleague- and child-centred account of relational anchoring rather than an equally weighted account of all three relationship types. Nurses described seeking informal consultation, sharing concerns with trusted peers, and drawing strength from the therapeutic relationship with patients.

*“When I am unsure, I talk to a colleague I trust. Just saying it out loud helps me think more clearly.” *

*(N10, Pediatric Medical, 13 years) *

The relationship with the child also served as an anchor, providing emotional motivation to persist through ambiguity.

*“When I look at the child’s face, I remember why I am here. The uncertainty does not disappear, but it becomes manageable.” *

*(N15, Pediatric Oncology, 14 years) *

Participants who described strong team cohesion reported greater confidence in managing uncertainty collectively.

*“When the whole team is uncertain together, it feels less frightening. We support each other, share what we observe, and make decisions as a group.” *

*(N3, Pediatric Medical, 7 years)*

##### Constructing Meaning Through Caregiving

Beyond relational strategies, nurses described constructing meaning from their work as a way of tolerating uncertainty. Rather than viewing ambiguity as a failure of knowledge, several participants reframed it as an inherent aspect of compassionate care.

*“I have learned that not knowing is part of the job. It does not mean I am a bad nurse. It means I am honest about what medicine can and cannot do.”*

*(N13, Pediatric Surgical, 8 years)*

This reframing allowed nurses to maintain professional identity and self-efficacy despite persistent clinical ambiguity.

*“I focus on what I can do: comfort the child, support the family, be present. That is enough, even when I do not have all the answers.” *

*(N1, Pediatric Medical, 10 years)*

Several participants described personal growth through their experiences with uncertainty, developing greater humility, patience, and emotional depth.

*“This work has changed me as a person. I am more patient, more humble. I have learned that uncertainty is part of life, not just work.”*

*(N14, Pediatric Oncology, 12 years)*

Together, these themes reveal coping with uncertainty as a multidimensional experience encompassing cognitive navigation, emotional endurance, and meaning-making. Pediatric nurses caring for children with complex chronic conditions engaged in sustained interpretive and relational work to maintain professional functioning in the face of pervasive clinical ambiguity.

Read hermeneutically rather than as discrete categories, these three themes describe a single interpretive movement: nurses first encounter uncertainty as an epistemic condition imposed by the child’s illness (Navigating the Unknown), then live it as an emotional and moral condition imposed on the self (The Emotional Weight of Not Knowing), and finally take it up as something to be inhabited and given meaning rather than resolved (Finding Footing in Uncertainty). This movement suggests that, within this sample, uncertainty tolerance in pediatric chronic illness nursing is not primarily a cognitive skill to be trained in isolation, but a relational and existential stance that develops through sustained exposure, professional relationships, and the reframing of not-knowing as intrinsic to compassionate care rather than as a personal or clinical failure. Not all participants described this trajectory with equal emphasis, and the account presented here foregrounds the shared structure across interviews rather than claiming an identical experience for every nurse.

## 4. Discussion

This study explored how pediatric nurses experience and cope with uncertainty when caring for children with complex chronic conditions. The findings demonstrate that clinical uncertainty in this context is not a transient challenge but a persistent experiential condition that shapes cognitive processing, emotional well-being, and professional identity. Nurses described uncertainty as layered, encompassing diagnostic ambiguity, prognostic unpredictability, and the moral complexity of acting with incomplete information. Importantly, coping was not passive but involved active relational and existential strategies, and more limited cognitive strategies, that enabled sustained professional engagement. The principal conceptual contribution of this study is to reframe uncertainty tolerance in pediatric chronic illness nursing as a relational and existential accomplishment sustained through colleague and patient relationships and through meaning-making, rather than as a purely intrapsychic or trainable cognitive skill, extending Mishel’s largely intrapsychic model of uncertainty appraisal into a professional, relationally embedded register.

### 4.1. Interpretation in Relation to Existing Literature

The findings align with and extend Mishel’s Uncertainty in Illness Theory [7], which identifies ambiguity, complexity, inconsistency, and unpredictability as core antecedents of illness-related uncertainty. While Mishel’s framework was originally developed for patients and family caregivers, participants in the present study described analogous cognitive appraisal processes, including difficulty structuring clinical information, identifying meaningful patterns, and predicting outcomes. The present study extends this framework by foregrounding the sustained nature of uncertainty in chronic illness nursing, consistent with evidence that unfamiliar symptom patterns and limited structural support amplify rather than resolve illness-related uncertainty [25].

The emotional burden described by participants resonates with literature on moral distress in pediatric nursing. Previous research has documented that nurses experience moral distress when institutional or clinical constraints prevent them from acting in accordance with their professional judgement [26,27]. Our findings add nuance by identifying uncertainty itself as a generator of moral tension, distinct from but intersecting with moral distress. The conflict between the imperative to act and the recognition that premature action may cause harm represents a specific form of professional suffering that warrants targeted support.

The relational coping strategies identified in this study are consistent with broader evidence on healthy nurse work environments, including the role of collegial support, and with literature on nursing resilience [28,29]. Peer consultation, team cohesion, and therapeutic relationships with patients have been previously identified as protective factors against burnout and compassion fatigue. However, the present findings highlight the particular importance of relational anchoring in contexts of sustained uncertainty, where the absence of definitive clinical knowledge increases nurses’ reliance on interpersonal resources for cognitive and emotional regulation.

The theme of constructing meaning through caregiving resonates with literature on the existential dimensions of suffering in serious illness, and with nurses’ moral resilience and professional identity [30,31]. Participants who reframed uncertainty as intrinsic to compassionate care, rather than as evidence of professional inadequacy, reported greater resilience and sustained motivation. This finding aligns with research on tolerance of ambiguity as a professional competency and supports educational initiatives that cultivate uncertainty tolerance alongside clinical knowledge [32,33].

Cultural and institutional contexts shaped participants’ experiences in ways consistent with literature on nursing practice in Saudi Arabia [34]. The rapid transformation of the healthcare system under Vision 2030, combined with cultural expectations surrounding childhood illness and family involvement, plausibly created additional layers of complexity for nurses navigating uncertainty; however, participants’ accounts, reported in Section 3.2, spoke more directly to relational and emotional coping within units than to these system-level or cultural factors specifically, and this interpretation should therefore be read as a contextual inference rather than a directly evidenced finding. With this caveat, these contextual factors point to a need for culturally sensitive support strategies and institutional frameworks that acknowledge uncertainty as a legitimate professional challenge.

### 4.2. Implications for Practice, Education, and Policy

The findings have several implications for pediatric nursing practice. First, institutions should develop formal recognition that clinical uncertainty is an inherent feature of chronic illness care rather than a deficiency to be eliminated. Creating structured opportunities for nurses to discuss uncertainty, such as regular reflective practice sessions or Schwartz Center Rounds, may be a candidate approach for future evaluation to reduce emotional isolation and strengthen collective coping capacity; as these models were developed largely in Western healthcare settings, their acceptability and format may need adaptation for the professional hierarchies, language mix, and workplace norms of Saudi tertiary hospitals before implementation.

Second, responsibility for managing uncertainty should not rest with individual nurses alone: nursing education programmes could integrate instruction on uncertainty tolerance, reflective practice, and communication skills for discussing ambiguity with families and colleagues, but these individual-level measures should be paired with organisational measures, such as adequate staffing and skill mix, clearer channels for raising concerns across professional hierarchies, and structured intra-team communication, since the findings suggest uncertainty was experienced, and best managed, relationally rather than by individual nurses working alone. Simulation-based education that incorporates scenarios without clear diagnoses or definitive interventions may be one component of this broader approach for future evaluation.

Third, interdisciplinary team structures should foster collaborative decision-making in uncertain clinical situations. Encouraging shared responsibility for clinical judgements in ambiguous cases may reduce the moral burden experienced by individual nurses and promote patient safety through collective vigilance.

At a policy level, healthcare organisations serving pediatric populations with complex chronic conditions could consider investing in psychological support services tailored to the unique stressors of chronic illness nursing. Peer support programmes, clinical supervision models, and access to counselling are proposed here as practice-informed recommendations warranting future evaluation, rather than as interventions whose effectiveness this study has tested, that may help mitigate cumulative emotional strain and support workforce retention.

### 4.3. Strengths and Limitations

This study provides contextually grounded insight into how pediatric nurses experience and navigate uncertainty in chronic illness care within tertiary healthcare settings in Saudi Arabia, a region where qualitative research on nursing practice in pediatric chronic illness remains limited. The hermeneutic phenomenological approach enabled exploration of cognitive, emotional, and relational dimensions of coping that may not be captured through quantitative methodologies.

However, findings are limited to nurses working in tertiary hospitals within one geographic region, which may influence transferability to other healthcare systems or community-based settings. Additionally, self-reported experiences may be shaped by recall bias or social desirability. Several further limitations warrant note. Recruitment relied on self-selection following a manager-circulated invitation, and although nurse managers played no role in selecting or approaching participants, self-selection may still have favoured nurses with stronger views or greater comfort discussing uncertainty. A single interviewer conducted all interviews, which supports consistency of approach but means the findings also reflect that interviewer’s particular style and rapport with participants. Interviews were conducted either face-to-face or via secure video conferencing depending on feasibility, and possible differences in disclosure or rapport between these two modes were not formally assessed. Finally, given the multinational nursing workforce described in Section 2.1, all interviews were conducted in English, which was not the first language for most participants (Table 1); no translation was undertaken at any stage, but variation in participants’ English proficiency and idiom may still have shaped the depth and precision with which emotionally complex experiences were articulated, and how the research team interpreted these accounts. Reflexive journaling and team-based analytic discussion were used throughout to manage, rather than eliminate, the researcher’s own clinical background and pre-understandings; consistent with hermeneutic phenomenology, this reflexive engagement is treated here as a feature of the interpretive process rather than solely as a limitation to be minimised. Future research across diverse settings and incorporating longitudinal or observational methods may deepen understanding of how coping with uncertainty evolves over nurses’ careers.

## 5. Conclusions

Grounded in the accounts of 15 nurses working in tertiary hospital pediatric units in the Riyadh region, this study offers an interpretive, rather than broadly generalisable, account of coping with clinical uncertainty as a dynamic interplay of relational and existential meaning-making, alongside more circumscribed cognitive navigation, in pediatric chronic illness care. Uncertainty is experienced not as a transient challenge but as a persistent condition that shapes professional identity, emotional well-being, and clinical practice. Pediatric nurses develop sophisticated relational and existential strategies to sustain their practice, yet require greater institutional recognition and support. These findings should be read as reflecting the particular institutional and cultural setting of this study rather than as claims applicable to pediatric nurses in general. Future research should examine interventions that strengthen uncertainty tolerance, promote reflective practice, and formalise peer support structures within pediatric care settings.

## Figures and Tables

**Table 1:** Socio-demographic and professional characteristics of participants (*n* = 15).

Socio-Demographic Characteristics	Frequency	Percentage
Age, mean (SD)	32.4 (5.1) years	—
Gender		
Male	4	26.7%
Female	11	73.3%
Nationality		
Saudi	5	33.3%
Non-Saudi	10	66.7%
First language		
Arabic	5	33.3%
Other languages	10	66.7%
Educational level		
Bachelor’s degree	11	73.3%
Postgraduate degree	4	26.7%
Total nursing experience, mean (SD)	8.7 (4.5) years	—
Pediatric nursing experience, mean (SD)	5.9 (3.2) years	—
Years worked in Saudi Arabia		
<5 years	5	33.3%
5–10 years	7	46.7%
>10 years	3	20.0%
Participating hospital		
Hospital A	8	53.3%
Hospital B	4	26.7%
Hospital C	3	20.0%
Clinical unit type		
Pediatric Medical	6	40.0%
Pediatric Surgical	4	26.7%
Pediatric Oncology	3	20.0%
Mixed Pediatric	2	13.3%

Note: Nationality and first language are reported as broad Saudi/non-Saudi and Arabic/other-language categories, and the participating hospital is reported using non-identifying labels (Hospital A, B, C), to maintain participant confidentiality and avoid disclosure of potentially identifiable demographic or institutional characteristics within this relatively small sample.

**Table 2:** Themes, sub-themes, and descriptions.

Theme	Sub-Theme	Description
Section 3.2.1. Navigating the Unknown	Living with Diagnostic Ambiguity	Nurses described ongoing uncertainty about diagnoses, prognoses, and care trajectories in children whose conditions defied clear clinical categorisation.
	Unpredictable Illness Trajectories	Children’s conditions fluctuated without warning, creating cycles of hope and apprehension that complicated clinical planning.
Section 3.2.2. The Emotional Weight of Not Knowing	Carrying the Burden Silently	Nurses internalised emotional distress arising from uncertainty, often feeling unable to express vulnerability in professional settings.
	Moral Tension Between Action and Restraint	Uncertainty generated moral conflict between the imperative to act decisively and the recognition that premature action could cause harm.
Section 3.2.3. Finding Footing in Uncertainty	Relational Anchoring	Relationships with colleagues and children, and to a lesser extent families, provided emotional grounding and practical guidance during periods of clinical ambiguity.
	Constructing Meaning Through Caregiving	Nurses created professional meaning by reframing uncertainty as intrinsic to compassionate, person-centred care rather than as a deficiency in knowledge.

## Data Availability

The qualitative datasets analysed during the current study are not publicly available due to confidentiality agreements with participants but are available from the corresponding author upon reasonable request.

## Supplementary Materials

The following supporting information can be downloaded at https://www.mdpi.com/article/10.3390/healthcare14152392/s1. Supplementary File S1: Semi-structured interview guide. Supplementary File S2: Completed COREQ (Consolidated Criteria for Reporting Qualitative Research) 32-item checklist.

## Abbreviations

The following abbreviations are used in this manuscript:

COREQ	Consolidated Criteria for Reporting Qualitative Research
IRB	Institutional Review Board
ORF	Ongoing Research Funding Program
