# Supplementary material for: Navigating Uncertainty in Pediatric Chronic Illness Care: A Hermeneutic Phenomenological Study of Nurses in Saudi Arabia
Source: Healthcare (Basel). 2026 Aug 4;14(15):2392. doi: 10.3390/healthcare14152392 (PMC13464106; doi:10.3390/healthcare14152392)
Supplement: Supplementary file 1 [file healthcare-14-02392-s001.zip › Supplementary File S1.pdf]

Supplementary File S1

Semi-Structured Interview Guide

Introduction

Thank you for agreeing to participate in this interview.

Section 1. Professional Background (Introductory Questions)

1. Could you tell me a little about your professional experience in pediatric nursing?

Prompts:

- Years of nursing experience …………………………..
- Current unit or department ……………………………
- Current professional role ……………………………...

2. What types of children with complex chronic conditions do you most commonly care for in your clinical practice?

Section 2. Experiences of Caring for Children with Complex Chronic Conditions

3. How would you describe your overall experience of caring for children with complex chronic conditions?

4. What aspects of providing nursing care for these children do you find most challenging?

5. Do you feel that caring for children with complex chronic conditions differs from caring for children with acute or short-term illnesses? If so, in what ways?

Section 3. Clinical Uncertainty and Decision-Making

6. Can you describe a situation in which you experienced uncertainty while caring for a child with a complex chronic condition?

Prompts:

- What contributed to the uncertainty?
- How did the situation develop?
- How did you respond?

7. How does uncertainty regarding disease progression, treatment response, prognosis, or clinical decision-making influence your nursing practice?

8. How do you make nursing decisions when information is limited or when the child's condition is particularly complex?

Prompts:

- What factors influence your decisions?
- Who do you consult?
- What resources do you rely on?

Section 4. Emotional and Ethical Dimensions

9. How does caring for children with complex chronic conditions affect you emotionally?

10. Have you encountered any ethically challenging situations while caring for these children?

Prompt:

- If appropriate, could you describe an example?

11. How do you cope with feelings of stress, anxiety, sadness, or emotional burden associated with caring for these children?

Section 5. Relationships with Families and the Multidisciplinary Team

12. How would you describe your interactions with the families of children with complex chronic conditions?

13. What challenges do you encounter when communicating with families, particularly when there is uncertainty about the child's condition or prognosis?

14. How do you collaborate with the multidisciplinary healthcare team (e.g., physicians, allied health professionals, and fellow nurses) when caring for these children?

Prompts:

- Communication
- Shared decision-making
- Team support

Section 6. Coping Strategies and Organizational Support

15. What strategies do you use to cope with the demands and pressures of caring for children with complex chronic conditions?

16. Do you feel that you receive adequate professional, organizational, or psychological support within your workplace? Why or why not?

17. What types of education, training, or workplace support do you believe would better prepare pediatric nurses to care for children with complex chronic conditions?

Section 7. Professional Reflection and Recommendations

18. How have these experiences influenced your professional identity as a pediatric nurse?

19. In your opinion, what changes could be made to improve care for children with complex chronic conditions and better support pediatric nurses?

20. Is there anything else you would like to share that you believe is important for understanding your experiences?

Closing the Interview

Thank you very much for taking the time to participate and for sharing your valuable experiences.

Before we conclude, **is there anything else you would like to add or discuss that we have not covered during this interview?**

Interviewer Notes
