# Supplementary material for: Navigating Uncertainty in Pediatric Chronic Illness Care: A Hermeneutic Phenomenological Study of Nurses in Saudi Arabia
Source: Healthcare (Basel). 2026 Aug 4;14(15):2392. doi: 10.3390/healthcare14152392 (PMC13464106; doi:10.3390/healthcare14152392)
Supplement: Supplementary file 1 [file healthcare-14-02392-s001.zip › Supplementary File S2.pdf]

Supplementary File S2
COREQ (Consolidated Criteria for Reporting Qualitative Research): 32-Item Checklist

No.	Item	Guide question	Reported where in manuscript
1	Interviewer/facilitator	Who conducted the interview?	§2.4, §2.6: conducted by the corresponding author
2	Credentials	What were the researcher's credentials?	§2.6: doctoral-level credentials of all analysis-team members
3	Occupation	What was their occupation at the time of the study?	§2.6: academic nurse with clinical experience in pediatric and critical care nursing
4	Gender	Was the researcher male or female?	§2.6: Male & female
5	Experience and training	What experience or training did the researcher have?	§2.6: scholarly background in qualitative and geriatric research; doctoral-level training
6	Relationship established	Was a relationship established prior to study commencement?	§2.2, §2.4: no member of the research team held a supervisory or evaluative role over participants; no prior clinical or personal relationship.
7	Participant knowledge of the interviewer	What did participants know about the researcher?	§2.4: participants were told about the researcher's role
8	Interviewer characteristics	What characteristics were reported about the interviewer/team (bias, assumptions, reasons/interests in the topic)?	§2.6: reflexive journaling; researcher's clinical background in pediatric/critical care nursing disclosed and examined for potential influence on interpretation
9	Methodological orientation and theory	What methodological orientation was stated to underpin the study?	§2.1: constructivist-interpretivist paradigm; hermeneutic (Heideggerian) phenomenology, per Alsaigh and Coyne
10	Sampling	How were participants selected?	§2.3: purposive, maximum-variation sampling
11	Method of approach	How were participants approached?	§2.4: indirect approach, study invitation circulated by email via nurse unit managers; interested nurses contacted the research team directly
12	Sample size	How many participants were in the study?	§2.3, §3.1, Table 1: n = 15
13	Non-participation	How many people refused to participate or dropped out? Reasons?	§2.3: 20
14	Setting of data collection	Where was the data collected?	§2.5: private, closed-door hospital room or secure encrypted videoconferencing

15	Presence of non-participants	Was anyone else present besides the participants and researchers?	§2.5: no one else present
16	Description of sample	What are the important characteristics of the sample?	§3.1, Table 1: age, gender, education, nursing/pediatric experience, unit type
17	Interview guide	Were questions, prompts, and guides provided by the authors? Was it pilot-tested?	§2.5, Supplementary File 1: guide developed for this study; pilot-testing procedure and refinements.
18	Repeat interviews	Were repeat interviews carried out?	§2.5: single interviews only; no repeat interviews
19	Audio/visual recording	Did the research use audio or visual recording to collect the data?	§2.5: audio-recorded with participant consent
20	Field notes	Were field notes made during and/or after the interview?	§2.5: field notes completed immediately after each interview
21	Duration	What was the duration of the interviews?	§2.5, §3.1: range 44–65 min; mean 53.2 (±6.1) min; total 798 min across the sample
22	Data saturation	Was data saturation discussed?	§2.3: information power framework (Malterud, Siersma and Guassora, 2016) used in place of a numerical saturation target, consistent with the study's hermeneutic-phenomenological design
23	Transcripts returned	Were transcripts returned to participants for comment/correction?	§2.5, §2.8: transcripts not returned for correction; a summary of preliminary themes was shared with a subset of participants for comment
24	Number of data coders	How many data coders coded the data?	§2.7: two members of the research team independently coded a subset of transcripts before reconciling
25	Description of the coding tree	Did authors provide a description of the coding tree?	§2.7: coding framework/codebook as a supplementary file
26	Derivation of themes	Were themes identified in advance or derived from the data?	§2.7: derived inductively through iterative hermeneutic-circle analysis (not identified in advance)
27	Software	What software was used to manage the data?	§2.7: qualitative data analysis software
28	Participant checking	Did participants provide feedback on the findings?	§2.8: a summary of preliminary themes was shared with a subset of participants for comment as part of credibility procedures
29	Quotations presented	Were participant quotations used to illustrate the themes/findings? Was each quotation identified?	§3.2: verbatim quotations presented for each theme and sub-theme

30	Data and findings consistent	Was there consistency between the data presented and the findings?	§3.2: consistent throughout
31	Clarity of major themes	Were major themes clearly presented in the findings?	§3.2, Table 2: three major themes presented
32	Clarity of minor themes	Is there a description of diverse cases or discussion of minor themes?	§3.2: sub-themes presented under each major theme
